# Catalytic conversion of diformylxylose to furfural in biphasic solvent systems

**DOI:** 10.3389/fbioe.2023.1146250

**Published:** 2023-02-10

**Authors:** Lizhen Huang, Zelun Bian, Dalin Li, Xin Cheng, Xiaolin Luo, Li Shuai, Jing Liu

**Affiliations:** ^1^ College of Materials Engineering, Fujian Agriculture and Forestry University, Fuzhou, China; ^2^ National Forestry and Grassland Administration Key Laboratory of Plant Fiber Functional Materials, Fuzhou, China; ^3^ Jiangsu Provincial Key Laboratory of Pulp and Paper Science and Technology, Nanjing Forestry University, Nanjing, China

**Keywords:** furfural, diformylxylose, xylose, biphasic system, kinetics, fractionation

## Abstract

Biobased furfural is a sustainable alternative to petrochemical intermediates for bulk chemicals and fuel production. However, existing methods for the conversion of xylose or lignocelluloses in mono-/bi-phasic systems to furfural involve non-selective sugar isolation or lignin condensation, limiting the valorisation of lignocelluloses. Herein, we used diformylxylose (DFX), a xylose derivative that is formed during the lignocellulosic fractionation process with formaldehyde protection, as a substitute for xylose to produce furfural in biphasic systems. Under kinetically optimized conditions, over 76 mol% of DFX could be converted to furfural in water-methyl isobutyl ketone system at a high reaction temperature with a short reaction time. Finally, isolation of xylan in eucalyptus wood as DFX with formaldehyde protection followed by converting DFX in a biphasic system gave a final furfural yield of 52 mol% (on the basis of xylan in wood), which was more than two times of that without formaldehyde. Combined with the value-added utilization of formaldehyde-protected lignin, this study would enable the full and efficient utilization of lignocellulosic biomass components and further improve the economics of the formaldehyde protection fractionation process.

## Introduction

Furfural has been recognized as a versatile intermediate to synthesize chemicals (e.g., furfuryl alcohol and furoic acid), fuels (e.g., methylfuran and long-chain alkanes), and functional materials (e.g., furfuryl alcohol and furfural-acetone resins) (Lee and Wu, 2021). As a result, the annual global production of furfural is now close to one million tons, and its demand continues to increase (Jaswal et al., 2022). Although furfural could be commercially produced from raw materials with high xylose content such as corn cob, the production of furfural from lignocelluloses such as wood and corn stover is inefficient, which would restrict the economics and development of biorefining industries (Luo et al., 2020).

Brønsted acid-catalyzed dehydration of xylose in aqueous media is a practical method for furfural production (Kabbour and Luque, 2020). However, in addition to the dehydration reaction, Brønsted acid can also catalyze other side reactions such as furan ring-opening and furfural condensation, resulting in low furfural yield (20–40 mol%) (Weingarten et al., 2010). For this reason, a biphasic solvent system consisting of water and organic solvent that is not miscible with water, has been developed to alleviate such undesirable side reactions (Román-Leshkov et al., 2006). In general, the solubility of furfural in the organic phase (e.g., toluene and methyl isobutyl ketone) is much higher than that in the aquous phase while the mineral acid is more soluble in water (Lin et al., 2021). Therefore, the furfural formed in the aqueous phase could be immediately transferred to the organic phase *via in-situ* extraction, which would effectively alleviate the acid-catalyzed side reactions of furfural in the aqueous phase and thus increase the yield of furfural (30–70 mol%) (Román-Leshkov et al., 2006; Shuai and Luterbacher, 2016; Lin et al., 2021).

Although the biphasic solvent system is promising for conversion of xylose to furfural, isolating xylose from lignocelluloses *via* acid-catalyzed hydrolysis of hemicelluloses in aqueous media is not selective, which highly restricts the application of the pathway (Questell-Santiago et al., 2018). Since Brønsted acid can simultaneously catalyze the hydrolysis of hemicelluloses and the dehydration of monosaccharides, some studies attempted to directly convert hemicelluloses in lignocellulosic feedstocks (e.g., eucalyptus, and bagasse) to furfural by a one-pot method in biphasic solvent systems (Matsagar et al., 2017). However, in the one-pot conversion process, lignin presents negative effects on the xylose conversion and furfural formation (Lamminpää et al., 2015). Degradation products derived from cellulose and lignin may also complicate the product mixture, which would be not conducive to subsequent furfural separation (Daorattanachai et al., 2013). Moreover, acid can also trigger severe lignin condensation during the one-pot conversion process, significantly devaluing lignin in other refining processes such as hydrogenolysis (Shuai et al., 2016; Gong et al., 2022; Luo et al., 2022).

To overcome the inherent defects of the one-pot conversion method, a two-step method, i.e. separating xylose or its derivative from lignocellulose and then converting it into furfural, remains a more attractive strategy if the condensation of lignin and the selectivity of hemicellulose hydrolysis to xylose could be effectively controlled (Questell-Santiago et al., 2018; Kabbour and Luque, 2020; Jaswal et al., 2022). Based on the acetalization of hydroxyl groups of carbohydrates with formaldehyde, Shuai et al. (2016) developed an effective method to fractionate the main components from lignocelluloses, which could simultaneously produce uncondensed lignin and stabilized xylose derivatives, i.e., diformylxylose (DFX). The conversion of DFX to value-added products has rarely reported. Therefore, the study aimed to investigate the acid-catalyzed conversion of DFX to furfural in different solvent systems. We also examined the kinetic behavior of DFX to furfural and compared the fractionation methods with or without formaldehyde addition for furfural production. This study validated the feasibility of a two-step method for furfural production, which would improve the overall refining efficiency of lignocelluloses.

## Materials and methods

### Materials

Xylose (99%), paraformaldehyde (98%), n-hexadecane (99%), methyl isobutyl ketone (99%), dichloromethane (98%), tetrahydrofuran (98%), dimethyl sulfoxide (98%), furfural (98%) were purchased from Aladdin^®^ Chemicals (Shanghai City, China). Toluene (98%) and concentrated hydrochloric acid (37 wt%) were ordered from XiLONG SCIENTIFIC (Guangzhou City, China), while γ-valerolactone (98%) was obtained from Macklin Inc. (Shanghai City, China). The procedures that used to synthesize DFX were detailed in Supplementary Materials. Synthesized DFX was characterized by Gas Chromatography-Mass Spectrometry (GC-MS) (Supplementary Figure S1) and its purity was determined as 98% by GC measurement based on an effective carbon number (ECN) method (Scanlon and Willis, 1985). Eucalyptus powder (40–60 mesh) was provided by Fujian Qingshan Paper Co., Ltd. (Sanming City, China).

### Acid-catalyzed conversion of DFX

Brønsted acid-catalyzed conversions of DFX and xylose were performed with monophasic or biphasic solvent systems in a glass-lined stainless steel reactor. For example, for the conversion of DFX with a biphasic solvent system, DFX, an internal standard (i.e., n-hexadecane) and a magnetic stirrer were successively added into the reactor that had been pre-loaded with HCl aqueous solution and organic solvent. The reactor was pressurized with 2 MPa N_2_ and then heated to the reaction temperature for a fixed reaction time. After that, the reactor was immediately cooled to room temperature with cold water and the sample solution was transferred into a centrifuge tube. The reactor was washed with deionized water three times and the washing solution was also poured into the centrifuge tube. The mixed solution in the centrifuge tube was fully separated into two phases through centrifugation at 10,000 rpm for 15 min, in which the organic phase was directly sampled and analyzed by GC. The aqueous phase was further diluted, fixed to a specific volume in a volumetric flask, and then subjected to high-performance liquid chromatography (HPLC) analysis, respectively.

### Lignocellulose fractionation and partition coefficient measurements

The hydrolysis of xylan in eucalyptus to obtain xylose or DFX was conducted in the GVL-water mixture according to a reported fractionation method with or without formaldehyde addition (Shuai et al., 2016). The partition coefficient of DFX or furfural in a biphasic system was measured by dissolving it (250 mg) in the mixture of water (1 mL) and organic solvent (1 mL) at room temperature, and measuring its concentration in two phases *via* GC and HPLC methods mentioned below (Lin et al., 2021).

### Measurements of the ECNs of DFX and furfural

The ECNs of DFX and furfural were measured by a reported method that used n-hexadecane as an internal standard (Scanlon and Willis, 1985). First, DFX, furfural and n-hexadecane were dissolved in methyl isobutyl ketone to obtain a mother solution. This solution was further diluted by methyl isobutyl ketone into solutions with different concentration gradients. These solutions were measured by GC, and the ECNs of DFX and furfural were calculated as follows:

$$N_{0}=N_{is}\frac{A_{0}{\times C}_{is}}{A_{is}{\times C}_{0}}$$ (1)

where *C*_*is*_ and *C*_*0*_ are the concentrations of the internal standard and determinand (DFX or furfural) in prepared solutions; *A*_*is*_ and *A*_*0*_ are the peak area of the internal standard and determinand (DFX or furfural) measured by GC; *N*_*is*_ is the reported ECN, i.e., 16, of the internal standard (Scanlon and Willis, 1985).

Based on the Eq. 1, the ECNs of DFX and furfural for the solutions with different concentration gradients, and their averages were calculated and listed in Supplementary Table S1, which would be used to calculate the concentrations of DFX and furfural in reaction samples.

### Analytical methods

The concentrations of DFX, xylose, and furfural in the diluted aqueous phase or monophasic co-solvent (i.e., the miscible mixture of water and organic solvent) were quantitatively determined by HPLC (Shimazu LC-20A) based on the external standard method. The HPLC was equipped with a refractive index detector (RID) and a Shodex SUGARSH-1011 column (8 × 300 mm^2^) using a sulfuric acid aqueous solution with a pH value of 2.2 as a mobile phase. The flow rate of the mobile phase was 0.8 mL/min, while the detector temperature, column temperature, sample injection volume, and detection time were fixed at 35°C, 30°C, 3 μL, and 60 min. After the fractionation of eucalyptus, the concentration of xylose or DFX in the GVL-water mixture was also analyzed by this HPLC method.

Based on the ECN method (Scanlon and Willis, 1985; Shuai et al., 2016), the molar amounts of DFX and furfural in the organic phase were determined by GC (Techcomp SCION 436C) equipped with a flame ionization detector (FID) and a capillary column (Techcomp SCION-5) using N_2_ as carrier gas at 1.5 mL/min. The column temperature program was fixed as follows: held at 50°C for 5 min, heated from 50°C to 300°C at 10°C/min, and held at 300°C for 5 min. The inlet temperature, split ratio and injection volume for GC-FID measurements were fixed as 300°C, 100:1, and 1 μL. The molar amount of DFX or furfural in the organic phase was calculated as:

$$\frac{n_{1}N_{1}}{n_{2}N_{2}}=\frac{A_{1}}{A_{2}}$$ (2)

where n_1_ is the molar amount (mmoL) of the internal standard (i.e., n-hexadecane) added into the reactor; n_2_ is the molar amount (mmoL) of the product formed in the organic phase; N_1_ and N_2_ are the ECNs of the internal standard and product; A_1_ and A_2_ are the measured peak area of the internal standard and product in the GC-FID chromatogram.

Since no xylose was detected in the organic phase that was not miscible with water, the conversion of DFX and the yield of product (xylose or furfural) in biphasic phases were calculated as:

$$X_{DFX}=\frac{n_{DFX}-\left(n_{DFX-org}+C_{DFX-aqu}\times V\right)}{n_{DFX}}\times 100$$ (3)

$$Y_{xyl}=\frac{C_{xyl-aqu}\times V}{n_{DFX}}\times 100$$ (4)

$$Y_{fur-aqu}=\frac{C_{fur-aqu}\times V}{n_{DFX}}\times 100$$ (5)

$$Y_{fur-org}=\frac{n_{fur-org}}{n_{DFX}}\times 100$$ (6)

$$Y_{fur-tol}=Y_{fur-aqu}+Y_{fur-org}$$ (7)

$$S_{fur}=\frac{Y_{fur-tol}}{X_{DFX}}\times 100$$ (8)

where *X*_*DFX*_ is the conversion (mol%) of DFX after reaction; *n*_*DFX*_ is the initial molar amount (moL) of DFX added into the reactor; *n*_*DFX-org*_ and *n*_*fur-org*_ are the molar amounts (moL) of DFX and furfural measured in organic phase; *C*_*DFX-aqu*_, *C*_*xyl-aqu*_, *C*_*fur-aqu*_, and *V* are the concentration (mol/L) of DFX, xylose, and furfural measured in the aqueous phase, and the total volume (L) of the aqueous phase after dilution; *Y*_*fur-aqu*_ and *Y*_*fur-org*_ are the yield (mol%) of furfural obtained in the aqueous and organic phases; *Y*_*xyl*_ is the yield (mol%) of xylose in the aqueous phase; *Y*_*fur-tol*_ and *S*_*fur*_ are the total yield (mol%) and selectivity (%) of furfural in the two phases.

For the monophasic reaction system, the conversion of DFX and the yield of product (xylose or furfural) were calculated based on the analysis results of HPLC in water or miscible co-solvent mixture. In particular, the total concentration of DFX, xylose, or furfural in two phases used for subsequent kinetic analysis was approximately calculated according to its measured or calculated molar amounts (moL), and the mass and density of the biphasic solvents that were initially added to the reactor.

$$C_{tol}=\frac{n_{tol}}{0.001\times \left(m_{aqu}/\rho_{aqu}+m_{org}/\rho_{org}\right)}$$ (9)

where *C*_*tol*_ and *n*_*tol*_ are the total concentration (moL/L) and molar amounts (moL) of DFX, xylose, or furfural in two phases; *m*_*aqu*_ and *m*_*org*_ are the mass (g) of HCl aqueous solution and organic solvent initially added into the reactor; *ρ*_*aqu*_ and *ρ*_*org*_ are densities of the HCl aqueous solution and organic solvent.

The contents of main components in eucalyptus wood were analyzed by a reported two-step hydrolysis method (Sluiter et al., 2008), and the results were listed in Supplementary Table S2.

## Results and Discussion

### Development of a biphasic solvent system

**FIGURE 1:**
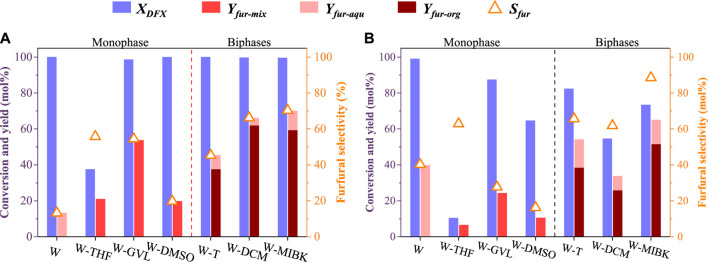
Conversion of DFX to furfural in different solvent systems at a HCl concentration and a reaction time of **(A)** 0.4 mol/L and 30 min and **(B)** 0.2 mol/L and 10 min. Other reaction conditions: DFX 0.5 g, 170°C, 2 g HCl aqueous solution and 8 g organic solvent, 400 rpm.

For Brønsted acid-catalyzed dehydration of monosaccharides (e.g., xylose and glucose) to furanics (e.g., furfural, and 5-hydroxymethylfurfural), previous studies reported that the solvation effect presents an important role in controlling the product yield and selectivity (Mellmer et al., 2014; Shuai and Luterbacher, 2016; Walker et al., 2018). By using HCl as a catalyst, three typical monophasic co-solvent systems, i.e., water-tetrahydrofuran (W-THF), water-γ-valerlactone (W-GVL), and water-dimethyl sulfoxide (W-DMSO), and three biphasic solvent systems, i.e., water-toluene (W-T), water-dichloromethane (W-DCM), and water-methyl isobutyl ketone (W-MIBK), were investigated for the conversion of DFX to furfural (Figure 1A). For comparison, an aqueous HCl solution (W) was also investigated as a control.

Under mild reaction conditions (10 min and 0.2 M H^+^), the conversion of DFX (99%, Figure 1B) in the W system was higher than that in other mixing solvent systems. This is mainly due to that the apparent concentration of hydrogen ions in water was diluted by organic solvent added into the monophasic or biphasic solvent system (Lin et al., 2021). For the monophasic or biphasic solvent systems, the W-GVL system achieved the highest DFX conversion (87%, Figure 1B). The reported molecular dynamics (MD) simulation results show that the solvation effects, i.e., adding co-solvent (e.g., GVL) to the acidic aqueous solution increases the local density of hydrated hydrogen ions near the reactant, is conducive to improving the hydrolysis efficiency of biomass-derived oxygenates such as cellobiose and ethyl tert-butyl ether (Walker et al., 2018). GVL may possess a similar solvation effect on the conversion of DFX in the monophasic co-solvent system (Mellmer et al., 2014). However, THF may exhibit the opposite solvation effect (Lee and Wu, 2021; Lin et al., 2021) because the conversion of DFX in W-THF system was much lower than those in other solvent systems under the same reaction conditions (Figure 1).

As the reaction conditions (30 min and 0.4 M H^+^) intensified, the DFX in all solvent systems except the W-THF system was almost completely converted (Figure 1A). However, the yield and selectivity of furfural in monophasic systems were lower than those in biphasic systems except the W-T system (Figure 1). This would be mainly caused by the severe condensation of furfural catalyzed by acid in the monophasic systems (Shuai and Luterbacher, 2016; Lee and Wu, 2021). For example, with the nearly complete conversion of DFX in the W system, increasing reaction time (10–30 min) and H^+^ concentration (0.2–0.4 mol/L) resulted in the decrease of furfural yield and selectivity from 40% to 13% (Figure 1). Based on the MD simulations, the solvation free energies (ΔG_sol_) of furfural in water-immiscible organic solvents such as toluene, MIBK, and DCM were reported to be much lower than that in water (Lin et al., 2021). As a result, most of the furfural formed in the aqueous phase could be quickly extracted into the organic phase, which would significantly reduce such adverse side reactions. For the biphasic systems, the ΔG_sol_ of furfural in MIBK and DCM were lower than that in toluene, making the extraction rate of the former towards furfural higher than the latter (Lin et al., 2021). In addition to other solvation effects such as solubility, this may be the key reason why the yield and selectivity of furfural in the W-MIBK and W-DCM systems were higher than those of W-T and monophasic systems (Lee and Wu, 2021; Lin et al., 2021).

**FIGURE 2:**
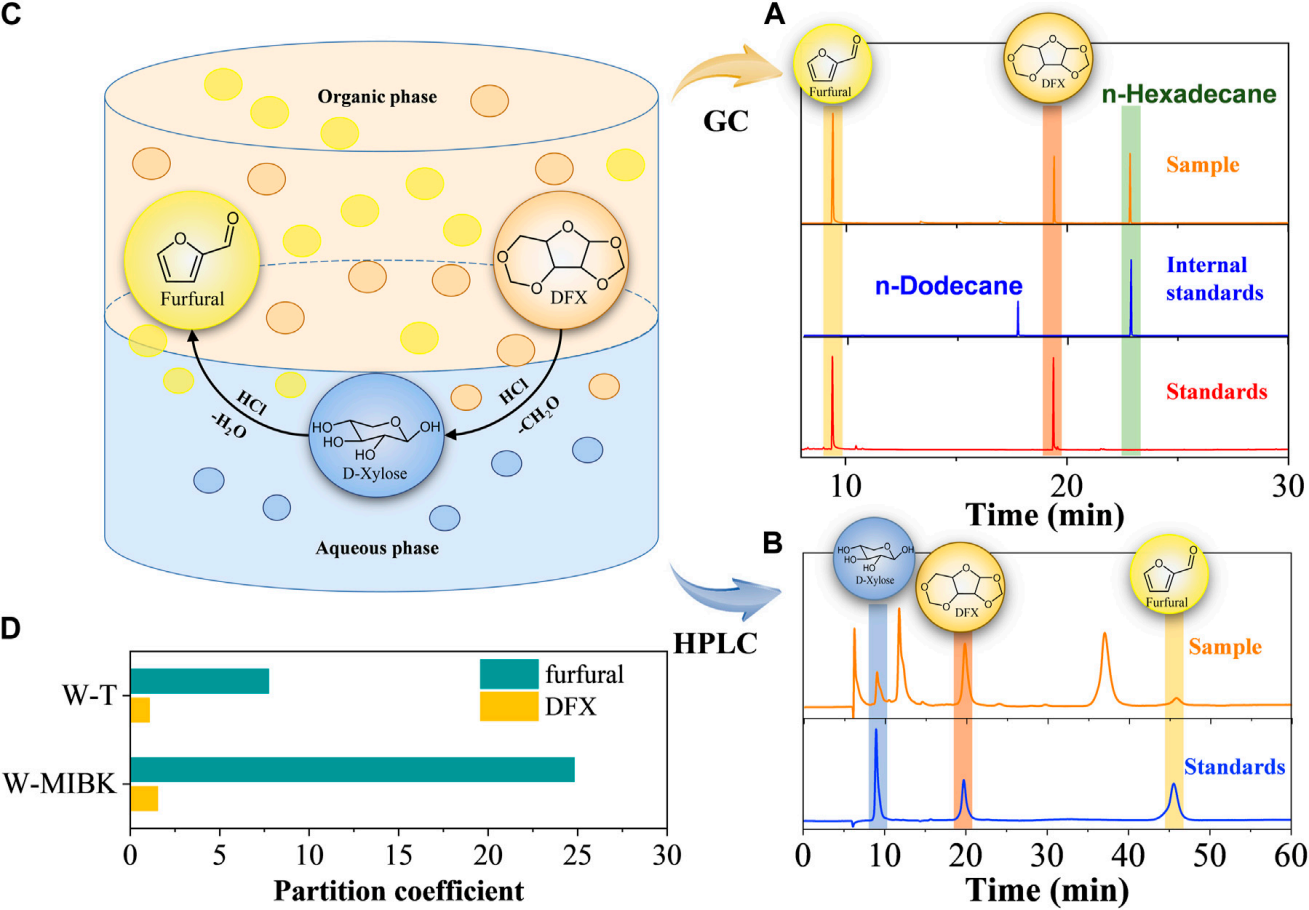
**(A)** GC and **(B)** HPLC analysis of samples and standards; **(C)** proposed transfer process of reactant and products in the biphasic system; **(D)** the partition coefficients of DFX and furfural in the biphasic system.

The transfer process of reactants and products in the two phases is useful to identify the influence of different biphasic systems on the conversion efficiency of DFX to furfural. Based on the chromatographies of the standards, only DFX and furfural were detected by GC in the organic phase (Figure 2A), whereas DFX, xylose, and little furfural were present in the aqueous phase (Figure 2B). Therefore, the conversion of DFX and the interphase transfer of furfural in the biphasic system could be simply described as follows (Figure 2C): 1) DFX was dissolved in the organic and aqueous phases according to its partition coefficient (Figure 2D). 2) Acid catalyzed the hydrolysis of DFX to xylose in the aqueous phase. When the concentration of DFX in the aqueous phase decreased, DFX was transferred from the organic phase to the aqueous phase. 3) In the aqueous phase, the acid further catalyzed the dehydration of xylose to furfural, which was rapidly extracted to the organic phase. However, acid-catalyzed furfural condensation competed with the *in-situ* extraction of furfural by organic solvents, which resulted in some unindentified compounds in the liquid chromatogram (Figure 2B).

In the above DFX conversion and furfural transfer process, it was found that in addition to the ΔG_sol_ effect, the partition coefficient of DFX ([DFX]_org_/[DFX]_aqu_) in the W-T system was slightly lower than that in W-MIBK system (Figure 2D), which would lead to relatively low solubility of DFX in toluene and the rapid conversion of DFX to xylose in the aqueous phase (Figure 1B). However, the partition coefficient of furfural ([furfural]_org_/[furfural]_aqu_) in the W-MIBK system was three times more than that of the W-T system (Figure 2D), which would contribute to the extraction of more furfural from the aqueous phase to the MIBK phase, thereby improving the final yield and selectivity of furfural (Figure 1) after complete conversion. Since the DFX conversion rate (Figure 1B) and furfural selectivity (Figure 1A) of W-MIBK system was slightly higher than those of W-DCM system, the biphasic W-MIBK system was investigated for the production of furfural from DFX.

### Conversion of DFX to furfural in W-MIBK system

**FIGURE 3:**
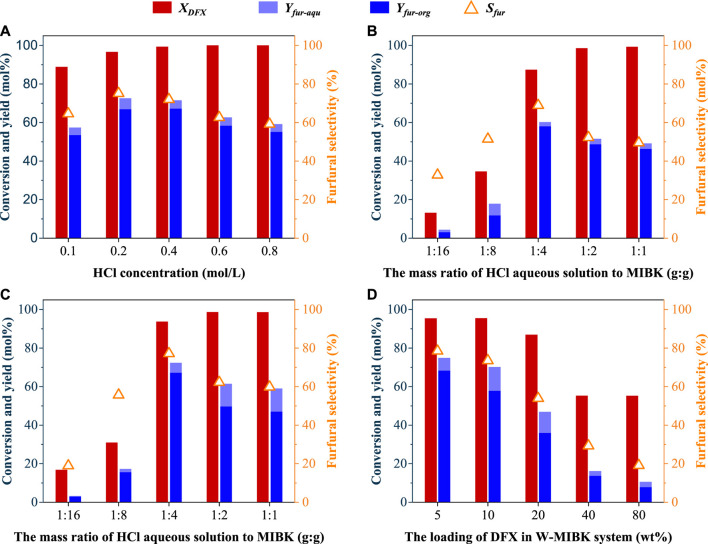
Effects of **(A)** acid concentration in aqueous phase, **(B)** and **(C)** solvent composition, and **(D)** DFX loading on the conversion efficiency of DFX to furfural in theW-MIBK system. The stirring speed and total mass of solvents for all reactions were 600 rpm and 10 g, the acid concentration used in Figures B∼D was 0.2 mol/L, and the DFX loading for Figure **(A–C)** was 5 wt%. A 1:4 (wt:wt) mass ratio of HCl aqueous solution to organic solvent was used for Figure **(A, D)**. Other reaction conditions of **(A)** and **(B)**: 170°C, 30 min; **(C, D)**: 185°C, 10 min.

Since the mass transfer efficiencies of reactant and products were reported to be much faster than their reaction rates (Weingarten et al., 2010), stirring speeds showed no obvious effects on the DFX conversion, and furfural yield and selectivity in aqueous solution (Supplementary Figure S2A). However, in addition to solvation effects (e.g., ΔG_sol_ and solubility), stirring is also an important factor affecting the transfer and conversion efficiencies of DFX and furfural in the biphasic system. Under the same reaction conditions (170°C, 30 min, and 0.4 M H^+^), stirring at 600 rpm was beneficial to improve the conversion of DFX from 82% to 99% and the yield of furfural from 37% to 72% in the W-MIBK system (Supplementary Figures S2B), compared with the reactions without stirring. Similar phenomena were also observed for W-T and W-DCM systems (Supplementary Figures S2C, D). Possibly due to the accelerated furfural condensation, further increasing the stirring speed led to a slight decrease in the yield and selectivity of furfural (Supplementary Figures S2). Besides, the concentration of HCl in the aqueous phase also affected the conversion and selectivity of DFX to furfural. When the concentration of HCl in the aqueous phase increased from 0.1 M to 0.2 M, the furfural yield increased from 57% to 73% (Figure 3A), indicating that high-concentration acid could improve the dehydration efficiency of DFX-derived xylose to furfural. However, the furfural yield and selectivity decreased from 73% to 59% after increasing the acid concentration to 0.8 M (Figure 3A).

Without changing the total mass of the two solvents, increasing the mass ratio of MIBK to HCl aqueous solution in the W-MIBK system would be beneficial to improve the extraction efficiency of furfural from aqueous phase to organic phase, which may favor increasing the final furfural yield and selectivity. As expected, with the increase of the mass ratio of MIBK to HCl aqueous solution from 1:1 to 4:1, the yield and selectivity of furfural increased gradually (Figures 3B, C). However, when the mass ratio of MIBK to HCl aqueous solution further increased to 16:1, the DFX conversion dramatically decreased from about 90% to less than 20%. For the production of furfural in the biphasic system using xylose as a substrate, xylose was only dissolved in the aqueous phase (Lin et al., 2021). Unlike xylose, DFX was soluble in both the aqueous and organic phases. Since the partition coefficient of DFX ([DFX]_org_/[DFX]_aqu_) in the W-MIBK system is greater than one (Figure 1D), increasing the MIBK proportion will be not conducive to the transfer of DFX from the organic phase to the aqueous phase, the hydrolysis of DFX to xylose as well as the dehydration reaction of xylose to furfural.

For W-MIBK system with a 4:1 mass ratio of MIBK to HCl aqueous solution, DFX loading did not show obvious effects on the DFX conversion as well as furfural selectivity when the loading of DFX in the biphasic system was within 10 wt% (Figure 3D). Unexpectedly, DFX conversion and furfural selectivity significantly decreased with the further increase in DFX loading, possibly due to the solubility limitation and intensified side reactions. Further explorations such as regulating the partition coefficients of reactants and products in a biphasic system would be necessary to clarify the reasons behind this adverse effect and to obtain high DFX-to-furfural efficiency at high DFX loading.

### Kinetic modeling of DFX conversion to furfural in the W-MIBK system

**FIGURE 4:**
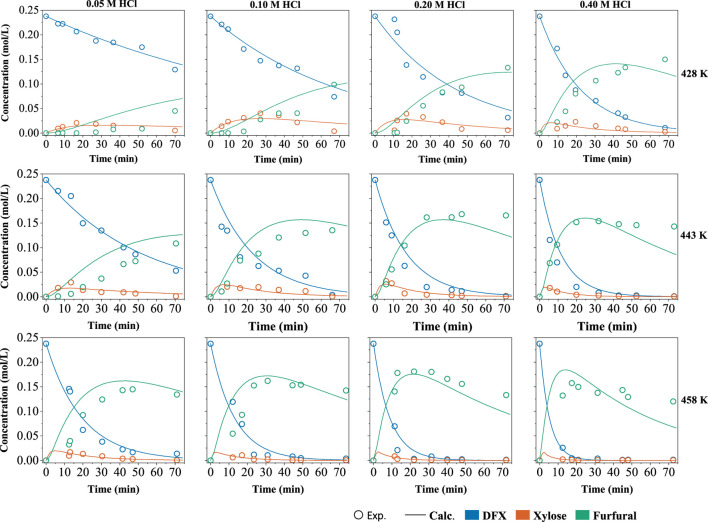
Measured concentrations of DFX, xylose and furfural in W-MIBK system and their kinetically predicted plots. Reaction conditions: DFX 0.5 g, 155–185°C, 0–74 min, 2 g HCl aqueous solution and 8 g MIBK, HCl 0.05–0.4 M, 600 rpm.

**SCHEME 1:**
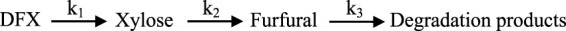
The reaction pathway of DFX in the biphasic system.

With a 4:1 (g:g) mass ratio for the W-MIBK system and a 5 wt% DFX loading, the kinetic modeling of DFX conversion to furfural was conducted at varied temperatures and HCl concentrations (Figure 4). Based on the products measured in two phases, the reaction pathway of DFX in the biphasic system was proposed in Scheme 1. It consists of the hydrolysis of DFX to xylose (reaction 1), the dehydration of xylose to furfural (reaction 2), and furfural degradation (reaction 3).

The reactions of 2 and 3 in biphasic systems are reported to be pseudo-first-order reactions (Weingarten et al., 2010). The disappearing trends of DFX as depicted in Figure 4 also coincides with the pseudo-first-order reaction. Thus, the overall rate equations for these three reactions can be written as:

$$\frac{{dC}_{DFX}}{dt}=-k_{1}C_{DFX}$$ (10)

$$\frac{{dC}_{xyl}}{dt}=k_{1}C_{DFX}-k_{2}C_{xyl}$$ (11)

$$\frac{{dC}_{fur}}{dt}=k_{2}C_{xyl}-k_{3}C_{fur}$$ (12)

where *C*_*DFX*_, *C*_*xyl*_, and *C*_*fur*_ are the total concentrations (mol/L) of DFX, xylose, and furfural in two phases; *k*_1_, *k*_2_, and *k*_3_ present the apparent rate constants of the reactions 1–3 in Scheme 1 (min^−1^); *t* is the reaction time (min) that considering the heating process (*t* = *t*_*reaction*_ + *t*_*heating*_/2) (Luo et al., 2013).

Based on the initial conditions (*t* = 0 min, $C_{DFX}=C_{DFX}^{0}$ ; $C_{xyl}=0$ mol/L, and $C_{fur}=0$ mol/L), the time-dependent expressions of *C*_*DFX*_, *C*_*xyl*_, and *C*_*fur*_ were integrated as:

$$C_{DFX}=C_{DFX}^{0}e^{-k_{1}t}$$ (13)

$$C_{xyl}=\frac{k_{1}C_{DFX}^{0}}{k_{2}-k_{1}}\left(e^{-k_{1}t}-e^{-k_{2}t}\right)$$ (14)

$$C_{fur}=\frac{k_{1}k_{2}C_{DFX}^{0}}{k_{2}-k_{1}}\left(\frac{e^{-k_{1}t}-e^{-k_{3}t}}{k_{3}-k_{1}}-\frac{e^{-k_{2}t}-e^{-k_{3}t}}{{k_{3}-k}_{2}}\right)$$ (15)

Based on the least squares method, the apparent rate constants of these kinetic equations were fitted in Table 1. As the fitted rate constants are a function of the acid concentration (Supplementary Figure S3), the Arrhenius equation can be modified as:

$$k=k_{0}{\left[C_{acid}\right]}^{\alpha }e^{-\frac{Ea}{RT}}$$ (16)

where *k*_*0*_ and *Ea* are the pre-exponential factor (min^−1^) and apparent activation energy (J/mol) of these kinetic models; *C*_*acid*_ is the concentration of acid in the aqueous phase (mol/L); *α* is the reaction order of acid; *R* and T are the ideal gas constant (J/mol/*K*) and the reaction temperature (*K*).

**TABLE 1:** The rate constants of the reactions 1–3 in Scheme 1 at different reaction temperatures and acid concentrations.

HCl concentration (mol/L)	Rate constants (min^−1^)								
	428 *K*			443 *K*			458 K		
	*k*_*1*_	*k*_*2*_	*k*_*3*_	*k*_*1*_	*k*_*2*_	*k*_*3*_	*k*_*1*_	*k*_*2*_	*k*_*3*_
0.05	0.007	0.086	0.006	0.019	0.207	0.008	0.052	0.485	0.010
0.10	0.014	0.077	0.008	0.043	0.307	0.009	0.078	0.925	0.011
0.20	0.022	0.137	0.010	0.058	0.392	0.012	0.113	1.306	0.015
0.40	0.045	0.383	0.014	0.087	0.852	0.017	0.190	2.116	0.019

For reactions 1 and 2 in Scheme 1, the rate constants of *k*_1_ and *k*_2_ were exponentially correlated with the concentration of acid in the aqueous phase (Supplementary Figures S3A, B). After logarithmic transformation, Eq. 16 was modified as

$$lnk=\ln k_{0}+\alpha {lnC}_{acid}-\frac{Ea}{R}\times \frac{1}{T}$$ (17)

However, the relationship between *k*_3_ and acid concentration was linear (Supplementary Figure S3C). As a result, the reaction order of acid for *k*_3_ was fixed as 1.0, and Eq. 16 was rearranged as

$$\ln\left(\frac{k}{C_{acid}}\right)=\ln k_{0}-\frac{Ea}{R}\times \frac{1}{T}$$ (18)

Based on the multivariate linear regression, the kinetic parameters (*k*_*0*_, *Ea*, and *α*) of Eqs.16‒18 were further fitted in Table 2. Based on these parameters, experimental results were well plotted against the predicted data (Supplementary Figure S4, *R* ^2^ > 0.92), showing good fits for these kinetic models towards the conversion of DFX to furfural and furfural degradation in the biphasic system.

**TABLE 2:** Pre-exponential factor, apparent activation energy, and the reaction order of acid for the developed kinetic models.

	*k*_*0*_ (min^−1^)	*Ea* (kJ/moL)	*α*
*k*_*1*_	1.17 × 10^10^	91.7	0.72
*k*_*2*_	1.87 × 10^13^	111.1	0.69
*k*_*3*_	2.44 × 10^5^	55.3	1.00

As shown in Table 2, the activation energies and reaction orders of acid for the reactions 1 and 2 in Scheme 1 are comparable, indicating that the sensitivities of these two reactions to the temperature and acid concentration would be similar. Non-etheless, for the reactions conducted at the same temperature and acid concentration, the rate constant of the reaction 1 is lower than that of the reaction 2 by nearly an order of magnitude (Table 1). Unlike the production of furfural using xylose as a substrate, the reaction 1 thus becomes the rate-determining step for the DFX-to-furfural conversion process in the biphasic system. This is mainly caused by the low partition coefficient of DFX ([DFX]_org_/[DFX]_aqu_) in the biphasic system (Figure 1D), which limits the overall hydrolysis rate of DFX to xylose. Because the solubility of mineral acid catalysts such as HCl in the MIBK phase is almost negligible. DFX can be catalyzed to xylose by the acid in the aqueous phase only when DFX in the organic phase is gradually transferred to the aqueous phase.

Since the activation energies and pre-exponential factors for the first two reactions are higher than those of the reaction 3 (Table 2), high reaction temperatures and short reaction times would be preferred for maximizing the furfural yield. Although increasing acid concentration favors improving the reaction rates (*k*_1_ and *k*_2_) of the reactions 1 and 2, furfural degradation (the reaction 3 with a *α* of 1.0) is more acid-dependent than the first two reactions (*α* < 1.0). As a result, a high acid concentration would decrease furfural selectivity (Figure 3A). According to the measured data and fitting plots (Figure 4), a maximum furfural yield of 76 mol% (Supplementary Figure S5) was eventually optimized at 185°C for 22 min with a moderate acid concentration of 0.2 M. This yield was two times that obtained in the monophasic aqueous solution (Figure 1), validating the distinct advantage of the biphasic system such as W-MIBK for converting DFX to furfural.

### Effect of fractionation process on the furfural production

**FIGURE 5:**
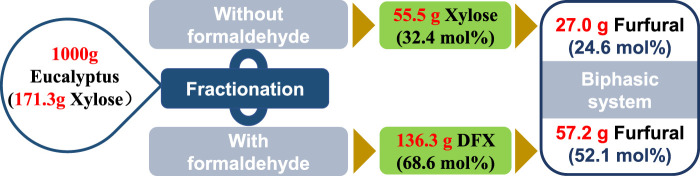
Comparsion of fractionation without or with formaldehyde addition for furfural production.

According to the previously-reported fractionation method (Shuai et al., 2016), the hydrolysis of xylan in eucalyptus to xylose or DFX was conducted in GVL-water (9:1, v/v) mixture without or with formaldehyde addition. For the fractionation with formaldehyde, 136.3 g of DFX was obtained from 1,000 g of eucalyptus wood that containing 171.3 g xylose (Figure 5). However, under the same fractionation conditions, the formaldehyde-free fractionation method only yielded 55.5 g of xylose. Assuming that the conversion efficiency of xylose in a biphasic system such as W-MIBK is the same as that of DFX (76 mol% furfural yield, Figure 4), the final furfural yield (on xylan in eucalyptus) for the formaldehyde-containing method was found to be twice that of the fractionation without formaldehyde (Figure 5). This discrepancy mainly lies in the acetalization of xylose with formaldehyde that can effectively inhibit the acid-catalyzed degradation of xylose during fractionation process (Shuai et al., 2016; Questell-Santiago et al., 2018).

Although the one-pot method can simplify the furfural production process (Matsagar et al., 2017), it inevitably causes the condensation of lignin under acidic conditions, devaluing the utilization efficiency of renewable aromatic carbon resources. With formaldehyde protection, Shuai’s study reported that lignin condensation could be effectively avoided in the formaldehyde-involved fractionation process (Shuai et al., 2016). Compared to one-pot furfural production methods (Matsagar et al., 2017; Kabbour and Luque, 2020; Jaswal et al., 2022), the two-step method consisting of formaldehyde-protected fractionation and biphasic conversion could not only obtain comparable furfural yield (52 mol%) but also provide an alternative strategy for the valorization of lignocelluloses.

## Conclusion

We have shown that the use of DFX as an alternative to xylose for furfural production could improve the utilization efficiency of xylan in lignocelluloses. Compared with aqueous solution and monophasic co-solvent systems, biphasic systems such as W-MIBK mixture enabled the efficient conversion of DFX to furfural, in which the conversion process mainly included the hydrolysis of DFX to xylose, the dehydration of xylose to furfural, and the transfer of furfural from the aqueous phase to the organic phase. In the biphasic W-MIBK system, we were able to obtain a decent furfural yield under kinetically optimized conditions. These results pushed forward the combination of the DFX conversion in the biphasic system and lignocellulosic fractionation that used formaldehyde to stabilize xylose *via* acetalization. As a result, such a combined two-step method achieved an overall furfural yield of 52 mol% based on the content of xylan in eucalyptus wood, which was almost two times the yield of the control process without formaldehdye addition. Because formaldehyde-stabilized fractionation also has the advantage of isolating highly active lignin, this combination approach could not only lead to the high conversion efficiency of xylan to furfural but also facilitate the integrated utilization of the three major biopolymers in lignocelluloses.

## Data Availability

The original contributions presented in the study are included in the article/Supplementary Material, further inquiries can be directed to the corresponding authors.
