# Supplementary material for: Catalytic conversion of diformylxylose to furfural in biphasic solvent systems
Source: Front Bioeng Biotechnol. 2023 Feb 10;11:1146250. doi: 10.3389/fbioe.2023.1146250 (PMC9950390; doi:10.3389/fbioe.2023.1146250)
Supplement: Supplementary file 1 [file DataSheet1.docx]

**Supplementary materials**

**Supplementary methods**

**Diformylxylose synthesis**

Diformylxylose (DFX) was synthesized according to reported procedures (Shuai et al., 2016; Questell-Santiago et al., 2018) with minor modifications. First, 4 L of 1,4-dioxane and 230 mL of concentrated hydrochloric acid (37 wt%) were sequentially added into a 10-L glass reactor and stirred for 5 min at 200 rpm. Then, 110 g of xylose and 175 g of paraformaldehyde were added to such reactor, and the mixture was heated to 80 °C and reacted for 1 h at a stirring speed of 200 rpm. At the end of the reaction, the reactor was cooled to room temperature in air. The pH value of resultant reaction solution was adjusted by sodium bicarbonate to 6‒7. Meanwhile, some anhydrous sodium sulfite was added to remove the peroxides in neutralized solution. The organic solvent and water in resulted solution were removed by vacuum drying at 60 °C, and yellow molten substances were obtained.

A mixture of ethyl acetate (500 mL) and deionized water (200 mL) was used to dissolve these molten substances. Resulted slurry was filtrated to remove insoluble substances. The organic solvent and water in collected filtrate were removed by vacuum drying at 60 °C, in which molten substances were re-formed. Such purification process was repeated three times. After that, anhydrous ethanol that heated to boiling was gradually added to dissolve the resulting molten substances to obtain a saturated solution. After cooled to room temperature, the saturated solution was further cooled in a refrigerator overnight at 4 °C for precipitating white crystals. The white crystals were vacuum dried at room temperature for 24 h, and powdery DFXs were finally obtained.

**Characterization of DFX by Gas Chromatography-Mass Spectrometry (GC-MS)**

Prior to GC-MS characterization, 10 mg of DFX was dissolved by 1 mL of chloroform in a 2-mL sample vial. DFX solution was analyzed by a GC-MS (Techcomp SCION 436SQ) equipped with an electron ionization (EI) ion source. For chromatography conditions of GC-MS, a capillary column (Techcomp SCION-5MS) that using helium gas as carrier gas at 1.5 mL/min was programed as follows: held at 40 °C for 8 min, heated from 40 °C to 300 °C at 10 °C/min, and held at 300 °C for 5 min. The temperature of EI ion source, split ratio, injection volume and solvent delay time for GC-MS measurement were fixed as 200 °C, 15: 1, 1 μL and 8 min.

**Figure S1** Synthesis and characterizations of DFX.

**Figure S2** Effects of stirring speed on the conversion efficiencies of DFX to furfural in (A) W; (B) W-MIBK; (C) W-T; (D) W-DCM systems. Other reaction conditions for biphasic systems: DFX 0.5 g, 170 °C, 30 min, 2 g HCl aqueous solution, 8 g MIBK, 0.4 M HCl in aqueous phase. For monophasic aqueous phase, 10 g of HCl aqueous solution was used, and other conditions were the same as the biphasic systems.


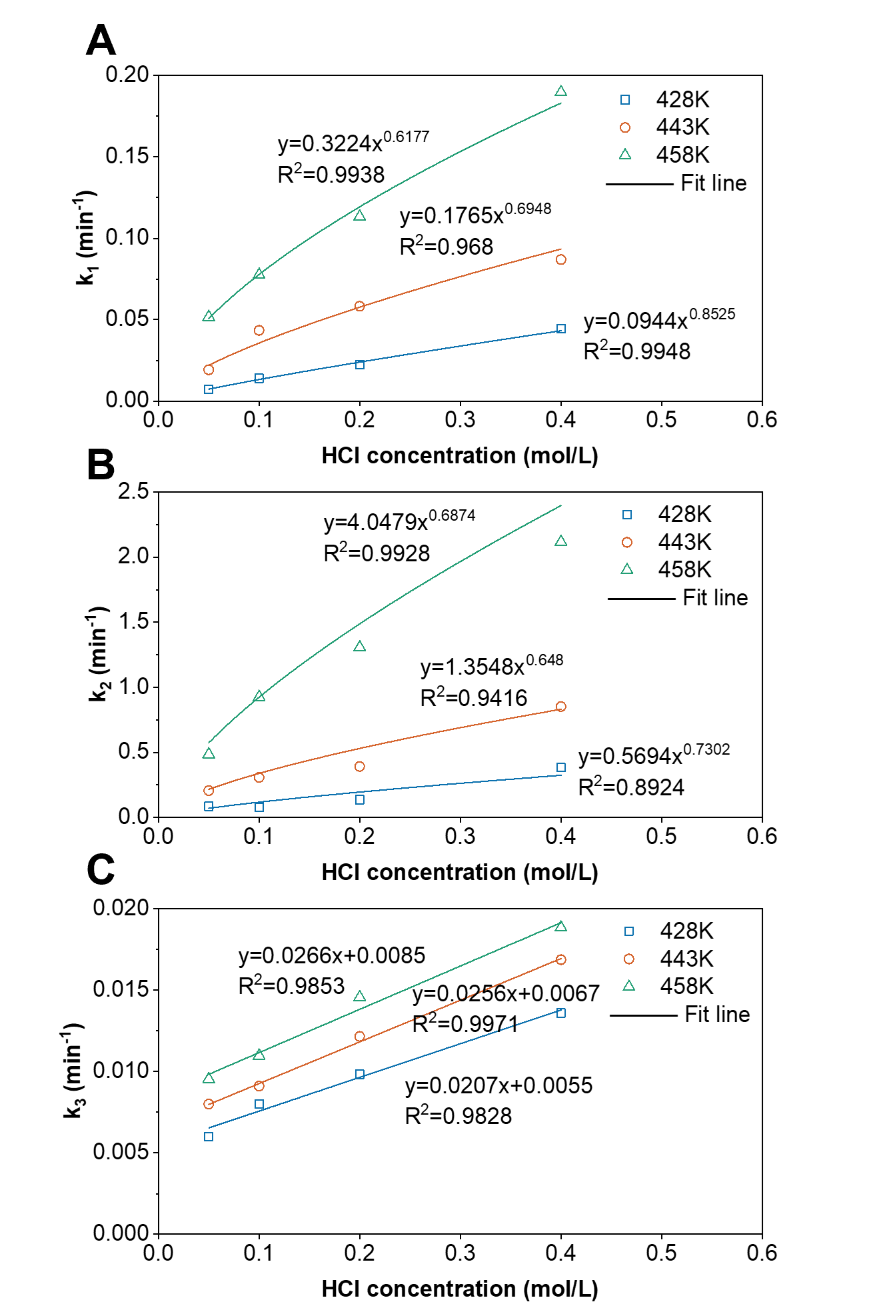


**Figure S3** The correlations of (A) *k_1_*, (B) *k_2_*, and (C) *k_3_* with the concentrations of HCl in aqueous phase under different reaction temperature.


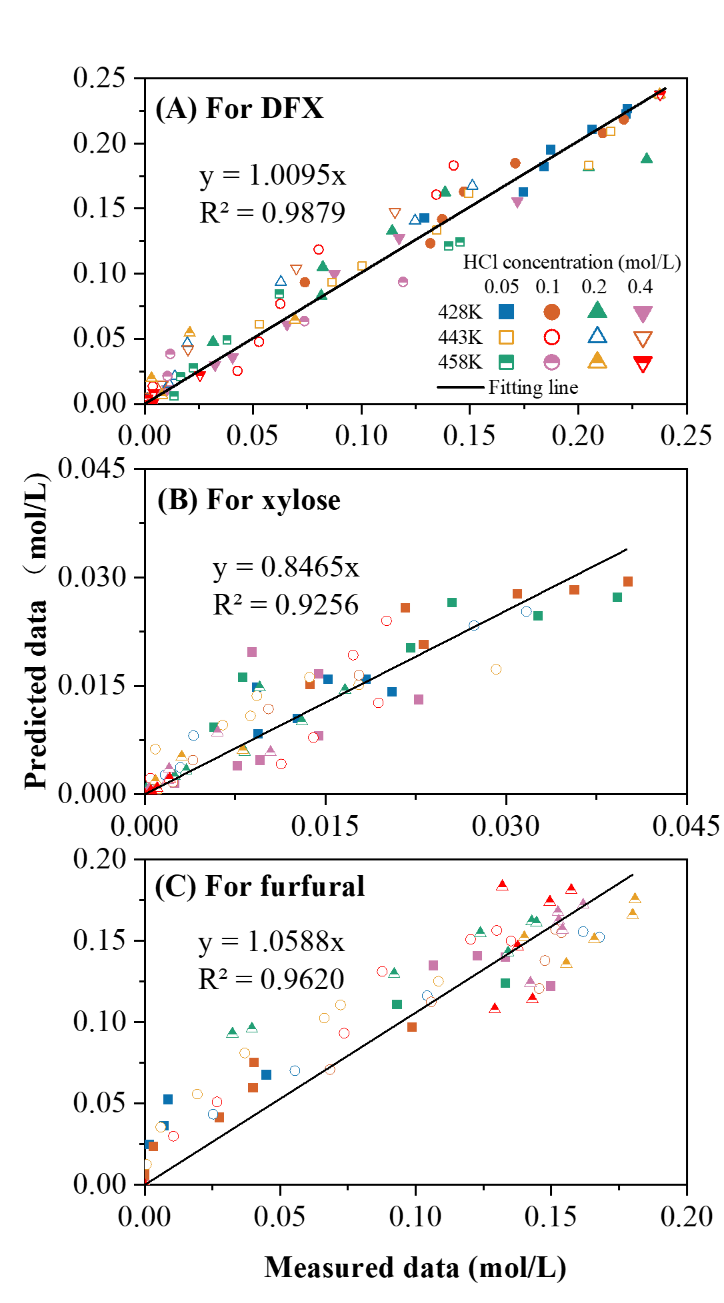


**Figure S4** The comparisons of experimentally measured (A) DFX, (B) xylose, and (C) furfural concentrations with the predicted data.


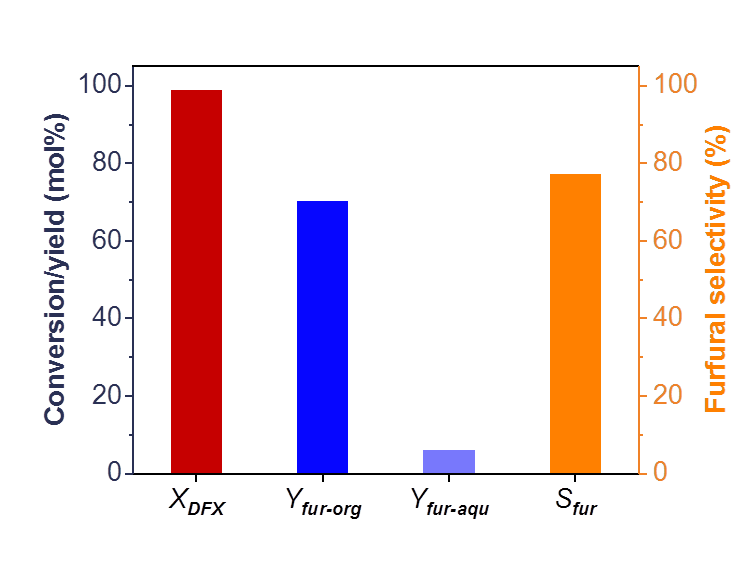


**Figure S5** DFX conversion, furfural yield and selectivity obtained under kinetically optimized conditions. Optimized reaction conditions: DFX 0.5 g, 185 °C, 22 min (*t* = *t_reaction_* + *t_heating_*/2), 2 g HCl aqueous solution and 8 g MIBK, HCl 0.2 M, 600 rpm.

Table S1 The ECNs of DFX and furfural for different prepared solutions and their averages

|  | | The concentration of determinand (DFX or furfural) in prepared solutions (g/L) | | | | | Average ECNs |
| --- | --- | --- | --- | --- | --- | --- | --- |
|  |  | 2 | 4 | 5 | 10 | 20 |  |
| Determinands | Furfural | 2.65 | 2.84 | 2.91 | 3.17 | 3.29 | 2.97 ± 0.26 |
|  | DFX | 3.15 | 3.30 | 3.35 | 3.56 | 3.57 | 3.39 ± 0.18 |

Table S2 The contents of components in eucalyptus wood

| Components | Contents (on o.d. wood, wt%) |
| --- | --- |
| Klason lignin | 25.56 |
| Acid soluble lignin | 3.02 |
| Glucan | 46.21 |
| Xylan | 15.16 |

**Supplementary References**

Questell-Santiago, Y.M., Zambrano-Varela, R., Talebi Amiri, M., and Luterbacher, J.S. (2018). Carbohydrate stabilization extends the kinetic limits of chemical polysaccharide depolymerization. *Nature Chemistry* 10(12)**,** 1222-1228. doi: 10.1038/s41557-018-0134-4.

Shuai, L., Amiri, M.T., Questell-Santiago, Y.M., Héroguel, F., Li, Y., Kim, H., et al. (2016). Formaldehyde stabilization facilitates lignin monomer production during biomass depolymerization. *Science* 354(6310)**,** 329-333. doi: 10.1126/science.aaf7810.
